# Age-related mesenchymal stromal cell senescence is associated with progression from MGUS to multiple myeloma

**DOI:** 10.1038/s41375-025-02621-7

**Published:** 2025-04-22

**Authors:** Natalya Plakhova, Vasilios Panagopoulos, Melissa D. Cantley, Laura J. Trainor, Duncan R. Hewett, Kimberley C. Clark, Jo Gardiner, Angelina Yong, Cindy Lee, Noemi Horvath, Peter I. Croucher, Dimitrios Cakouros, Sheila A. Stewart, Stan Gronthos, Andrew C. W. Zannettino, Krzysztof M. Mrozik, Kate Vandyke

**Affiliations:** 1 Myeloma Research Laboratory, School of Biomedicine, Faculty of Health and Medical Sciences, The University of Adelaide, Adelaide, SA Australia; 2 Precision Cancer Medicine Theme, South Australian Health & Medical Research Institute (SAHMRI), Adelaide, SA Australia; 3 Department of Haematology, Royal Adelaide Hospital, Adelaide, SA Australia; 4 Garvan Institute of Medical Research, Sydney, NSW Australia; 5 Mesenchymal Stem Cell Laboratory, School of Biomedicine, Faculty of Health and Medical Sciences, The University of Adelaide, Adelaide, SA Australia; 6 Department of Cell Biology and Physiology, ICCE Institute, Washington University School of Medicine, St. Louis, MO USA

**Keywords:** Myeloma, Cancer microenvironment

## Abstract

The risk of progression of monoclonal gammopathy of undetermined significance (MGUS) to multiple myeloma (MM) increases with advancing age, suggesting that progression may be influenced by age-related changes within the bone marrow (BM) microenvironment. We hypothesise that senescent mesenchymal stromal cells (MSCs), which accumulate in the BM with age, may contribute to MGUS progression to MM. Here, we show that, like BM MSCs from aged non-cancer controls, BM MSCs from both MM and MGUS patients exhibit a senescent phenotype characterised by enlarged, flattened morphology, increased β-galactosidase activity and *CDKN2A* expression, and decreased proliferation rate compared with BM MSCs from healthy young individuals. While coculture with BM MSCs suppresses the proliferative capacity of MM cell lines in vitro, induction of senescence via irradiation or replicative exhaustion in healthy MSCs relieves this suppression, compared with non-senescent MSCs. This may, in part, be attributable to upregulated expression of the BMP antagonist Gremlin1 in senescent MSCs, which facillitates MM cell proliferation. Notably, the risk of progression to MM was significantly elevated in MGUS patients with increased MSC senescence. Collectively, our data provide evidence that age-related accumulation of senescent MSCs may be a driver of MGUS to MM progression.

## Introduction

Multiple myeloma (MM) is an age-related cancer of plasma cells (PCs), with an average age at diagnosis of 70 years [1]. MM is universally preceded by the benign condition monoclonal gammopathy of undetermined significance (MGUS) [2], which is characterised by the presence of low levels of clonal PCs in the bone marrow (BM) in the absence of MM-defining features. For patients with non-IgM MGUS, the average yearly risk of progression to MM or related plasma cell disorders is 0.9%, with approximately 10% of all non-IgM MGUS patients developing MM during their lifetime [3, 4]. Notably, advanced age at follow up has been identified as a potential risk factor for MGUS to MM progression, with the risk of non-IgM MGUS progression to MM and other plasma cell disorders increasing from 0.4% patient years in younger patients (age <60 years) to 1.0% in older patients (age ≥60 years) [3]. Notably, increased age has been suggested to an independent risk factor for progression from MGUS to MM, even when corrected for other known risk factors including paraprotein level and serum free light chain (SFLC) level and ratio [4, 5].

The association between patient age and increased risk of progression from MGUS to MM [3, 5] suggests that age-related changes in the BM may create a supportive microenvironment that facilitates PC growth and disease progression. One of the key hallmarks of ageing is increased cellular senescence, which is induced by activation of cyclin-dependent kinase inhibitors, including p16^INK4A^, p14^ARF^, p21^Cip1^, that inhibit cell cycle progression [6]. Senescent cells are characterised by decreased proliferative capacity, flattened and enlarged morphology and increased activity of the enzyme β-galactosidase (β-gal). Cellular senescence is also associated with increased secretion of growth factors and pro-inflammatory cytokines, known as the senescence associated secretory phenotype (SASP) [7]. Notably, stromal cell senescence has been implicated in the development of various age-related cancers including breast and prostate cancer and certain leukaemias through secretion of pro-tumorigenic SASP factors [7, 8]. In MM, BM mesenchymal stromal cells (MSCs) provide critical support to PCs by increasing MM PC survival and resistance to chemotherapy [9]. To date, studies have demonstrated that MSCs from patients with MM have an increased capacity to support the growth and survival of MM cells, when compared with MSCs from healthy donors, in part due to upregulation of expression of key growth factors and cytokines in MM MSCs [10, 11]. MSC senescence is a feature of MM, with several studies reporting that MSCs from the BM of MM patients display higher levels of cellular senescence compared with those from healthy donors, as demonstrated by increased β-gal positivity, flattened and enlarged cell morphology, reduced proliferative and osteogenic differentiation potential and upregulated expression of senescence-associated genes including *CDKN2A* (p16/p14) and secretion of SASP factors [11–18]. However, it is unclear if senescent BM MSCs play a direct role in the development of MM.

The association of patient age with MGUS to MM progression and the importance of BM MSCs in MM pathogenesis suggests that BM MSC senescence may play a role in MM disease progression. In this study, we have, for the first time, comprehensively characterised the senescence-related phenotype of BM MSCs in both MGUS and MM patients and assessed the potential association between the level of MSC senescence and risk of MGUS to MM progression. In addition, we assessed the effect of senescent BM MSCs on MM cell growth in coculture and identified potential factors secreted by senescent MSCs that may facilitate MM cell outgrowth.

## Methods

### MSC cultures

Human sample collection and use for this project was approved by the Royal Adelaide Hospital Human Research Ethics Committee (HREC; protocol no. 940911a and 130114) and the Central Adelaide Local Health Network (CALHN) HREC (protocol R20110304 and R20181011). All individuals provided informed consent in accordance with the Declaration of Helsinki. Clinical data were provided by the Myeloma and Related Paraproteinaemias (MRP) Database (CALHN HREC reference no. 16351).

BM MSCs from healthy young volunteers, aged non-cancer individuals, or patients with MGUS or newly diagnosed, previously untreated MM were isolated by plastic adherence as previously described [19, 20] and cultured in α-modified minimum essential medium (αMEM) with 20% foetal bovine serum (FBS; Thermo Fisher Scientific) and 100 µM L-ascorbate-2-phosphate and supplements (complete αMEM). Cells were expanded to passage 3 and then sub-cultured twice per week (every 3–4 days), counted and population doublings were calculated using the following formula: population doublings=log_2_(cells harvested/cells seeded) [21]. MSC cultures were confirmed to fulfil the minimal criteria for defining human MSC [22] (Supplementary Fig. 1).

Primary mouse MSCs were isolated by plastic adherence from the tibiae and femora of eight-week-old and 18-month-old C57BL/KaLwRij.Hsd mice, as approved by the SAHMRI animal ethics committee (SAM448.19), and were cultured in complete αMEM. The generation of Gremlin1 over-expressing and empty vector (EV) mouse OP9 MSCs is previously described [23]. At passage 5, human BM MSCs were seeded in 96-well black-walled flat bottom plates (Corning Life Science) for coculture with MM PCs or in 6-well plates for assessment of β-gal activity and RNA isolation for qRT-PCR, as described in the supplementary methods. Where appropriate, MSCs were irradiated at 60 Gy prior to use in experiments.

### Gene expression analyses

Total RNA was extracted using TRIzol reagent (Invitrogen), treated with RQ1 DNAse (Promega) and reverse transcribed using SuperScript IV (Invitrogen). qRT-PCR was performed using primers listed in the supplementary methods. Gene expression was compared in human MSCs from *n* = 3 healthy controls and *n* = 4 MM donor MSCs using the publicly available microarray dataset GSE36474 [14], as previously described [24].

### Statistical analysis

Unless otherwise described, statistical analysis was performed using GraphPad Prism (version 9). Patient groups were compared with the nonparametric Kruskal-Wallis test with Dunn’s post-test or the Fisher’s exact test. Normalised qRT-PCR data were compared with the nonparametic Wilcoxon matched-pairs signed rank test. All other groups were compared with one-way ANOVA with Holm-Šídák’s post-test or paired or unpaired *t* tests, as appropriate. All tests were two-sided. Correlations were assessed using Pearson’s correlation co-efficient.

Progression to active MM was defined clinically as development of bone marrow plasmacytosis ≥10% and/or immunoglobulin ≥30 g/L with the development of CRAB features (hypercalcaemia, renal insufficiency, anaemia and bone lesions) [25] and/or initiation of therapy for myeloma disease. Patients were censored at the time of last clinical follow up (range: 84.7–512 weeks).

Receiver operating characteristic (ROC) analysis in IBM SPSS (version 28) was used to define cut-offs to optimize the sensitivity and specificity of the measure to differentiate progressing from non-progressing MGUS patients. Kaplan-Meier analysis was performed to analyse time-to-MM progression in MGUS patients, using the log-rank test to compare groups. Univariate and multivariable analyses of factors associated with progression to MM were conducted using SPSS. Initially, the proportional hazards assumption was tested, and univariate Cox regressions were conducted to identify factors that were significantly associated with progression to MM (*p* < 0.1). These factors were then included in a multivariable Cox proportional hazards model.

### Additional methods

See the Supplemental Material for additional material and methods.

## Results

### BM MSC senescence in MGUS patients is similar to that seen in MM patients and age-matched individuals without cancer

**Fig. 1:**
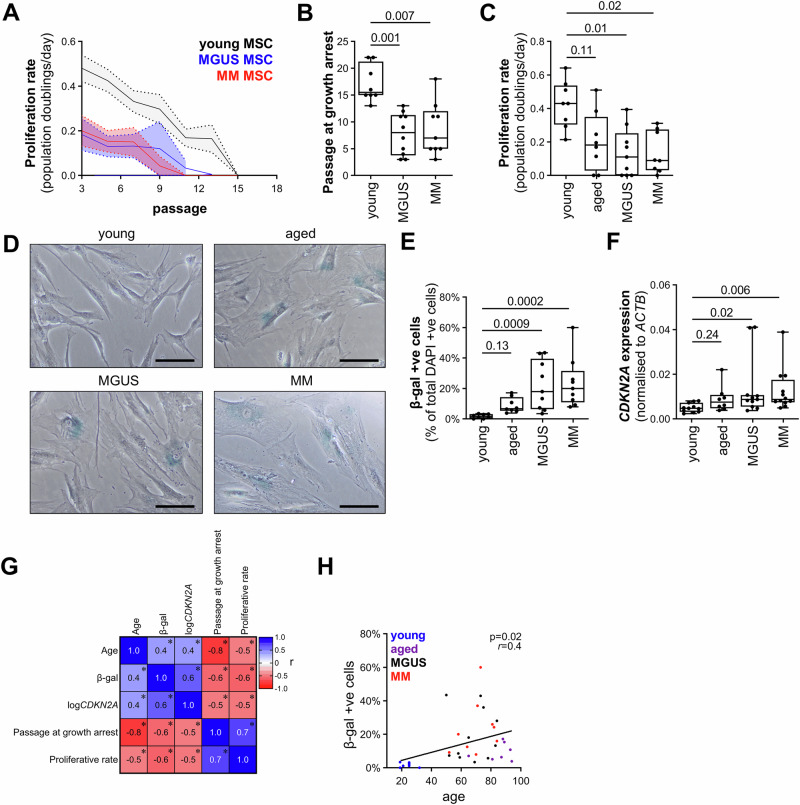
MSCs from MGUS and MM patients are characterised by an age-related senescent phenotype. **A** Human BM MSCs cultures were passaged twice weekly until cessation of proliferation. Once per week, cell proliferation rate (populations doublings per day) was assessed after 3 days in culture. Graph depicts the mean (solid line) and SEM (dotted lines) proliferation rate over sequential passage for young non-cancer controls (*n* = 8), MGUS (*n* = 10) or MM (*n* = 9) MSC donors. **B** Passage at which MSCs ceased proliferating is shown for young non-cancer controls (*n* = 8), MGUS (*n* = 10) or MM (*n* = 9) individual MSC donors. **C** MSCs from young (*n* = 8) or aged (*n* = 8) non-cancer controls and MGUS (*n* = 9) or MM (*n* = 8) patients at passage 5 were seeded and cell proliferation rate was assessed by conducting cell counts after 3 days in culture. **D** MSCs from young (*n* = 8) or aged (*n* = 8) non-cancer controls and MGUS (*n* = 9) or MM (*n* = 8) patients at passage 5 were seeded and stained for β-gal activity (blue) after 24 hours of culture. Representative images are shown; scale bar: 50 µm. **E** Percentage of β-gal-positive cells, as a proportion of total cells (identified by DAPI co-stain), was calculated for young (*n* = 8) or aged (*n* = 8) non-cancer controls and MGUS (*n* = 9) or MM (*n* = 8) patients. **F** Expression of *CDKN2A*, normalised to *ACTB*, was analysed by qRT-PCR in passage 5 MSCs from young non-cancer controls (*n* = 11), aged non-cancer controls (*n* = 8) and newly diagnosed MGUS (*n* = 11) and MM (*n* = 12) patients. **G** Correlation matrix (Pearson’s) showing association between donor age and senescent phenotype in MSCs from young and aged non-cancer controls and MGUS and MM patients. * indicates *p* < 0.05 for Pearson's correlation. **H** Scatter dot plot showing correlation between donor age and % β-gal positivity in MSCs from young and aged non-cancer controls and MGUS and MM patients. Box and whisker plots (**B**, **C**, **E**, **F**) depict median and interquartile ranges. *p* values are shown for Kruskal-Wallis test with Dunn’s post-test (**B**, **C**, **E**, **F**) or Pearson’s correlation (**H**).

While previous studies suggest that MM BM MSCs display increased levels of senescence compared with healthy donor MSCs [11–18], whether this is present at the MGUS stage, prior to development of MM, is unclear. Initially, we assessed the proliferation rate (population doublings/day) of BM MSCs from MGUS (*n* = 10; age: 48–76) and MM (*n* = 9; age: 52–86) patients and healthy young controls (*n* = 8; age: 18–32) weekly (every 2 passages) until they ceased proliferating. MSCs from MM and MGUS patients consistently exhibited a lower proliferation rate compared with MSCs from healthy young controls (Fig. 1A). MSCs from healthy young individuals continued to proliferate until at least passage 13 (median: passage 16; range: 13–22), while MSCs from MGUS and MM patients reached proliferative arrest as early as passage 3 (MGUS, median: passage 8; range: 3–13; MM, median: passage 11; range: 3–18; Fig. 1B).

Next, we compared the senescent phenotype of MSCs at passage 5 from MGUS (*n* = 9; age: 50–84) and MM patients (*n* = 8; age: 52–86), healthy young controls (*n* = 8; age: 17–32 years) and older non-cancer controls (*n* = 8; age: 65–94 years). The proliferation rate of MGUS and MM MSCs was significantly lower than that of young controls (*p* = 0.010 and *p* = 0.021, respectively; Kruskal-Wallis test with Dunn’s post-test; Fig. 1C) but did not significantly differ from that of older controls (both *p* > 0.99; Kruskal-Wallis test with Dunn’s post-test; Fig. 1C). MSCs isolated from MGUS and MM patients and older controls displayed an enlarged, flat, polygonal morphology characteristic of senescent cells which was not observed in those from younger controls (Fig. 1D). Senescence-associated β-gal staining revealed that while fewer than 5% of cells stained β-gal positive in MSC cultures from younger controls, β-gal positive cells were increased 10.6-fold in MGUS MSC cultures and 11.8-fold in MM MSC cultures (*p* = 0.0009 and *p* = 0.0002, respectively; Kruskal-Wallis test with Dunn’s post-test; Fig. 1E). Cultures from older controls were 4.0-fold higher than younger controls; however, this was not significantly different from younger controls (*p* = 0.13, Kruskal-Wallis test with Dunn’s post-test). Furthermore, expression of ageing-related replicative senescence marker *CDKN2A*, as assessed by qRT-PCR, was significantly increased 1.8-fold in MSCs from MGUS and MM patients, compared with younger non-cancer controls (*p* = 0.02 and *p* = 0.006, respectively, Kruskal-Wallis test with Dunn’s post-test; Fig. 1F). Notably, there were no statistically significant differences observed between MGUS patients, MM patients and older controls for any of the characteristics analysed.

We then investigated the association between donor age and senescent phenotype in MSCs from young and aged non-cancer controls and MGUS and MM patients. Donor age was found to be a strong predictor of the level of senescence observed in the MSC cultures, with age being positively associated with the percentage of β-gal positive MSCs (*p* = 0.020; Pearson’s *r* = 0.4 [95% CI: 0.07–0.6]) and *CDKN2A* expression (*p* = 0.013; Pearson’s *r* = 0.4 [95% CI: 0.09–0.6]), while there was an inverse correlation between age and MSC proliferation rate (*p* = 0.003; Pearson’s *r* = –0.8; 95% CI: –0.2 to –0.7) and passage at growth arrest (*p* < 0.0001; Pearson’s *r* = –0.5; 95% CI: –0.6 to –0.9) (Fig. 1G, H). Collectively, our data demonstrate, that the level of BM MSC senescence in MGUS patients is similar to that seen in MM patients and individuals without cancer of a similar age.

### BM MSC senescence predicts MM progression risk in patients with MGUS

**Fig. 2:**
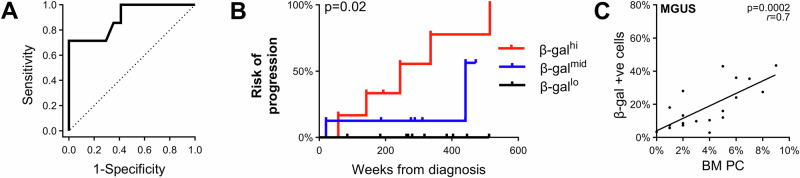
Increased MSC senescence in MGUS patients is associated with increased risk of MGUS to MM progression. **A** ROC curve analysis for MSC β-gal staining in MGUS patients, showing the sensitivity and specificity for distinguishing MGUS to MM progressors from non-progressing patients. **B** MGUS patients stratified as β-gal^hi^ (≥27.5% β-gal-positive MSCs; *n* = 6), MGUS patients stratified as β-gal^mid^ (10-27.5% β-gal-positive MSCs; *n* = 8) and β-gal^lo^ (<10% β-gal-positive MSCs; *n* = 10) were assessed for rate of MGUS to MM progression using Kaplan-Meier analysis. **C** Scatter dot plot showing correlation between % β-gal positivity in MSCs and bone marrow PC burden in MGUS patients. *p* values are shown for the log-rank test (**B**) or Pearson’s correlation (**C**).

Given MGUS patient age is positively associated with risk of progression to MM [3], we hypothesized that MSC senescence could be a driver of progression from MGUS to MM. In order to investigate this, we characterized the senescent phenotype in passage 5 MSCs from an extended cohort of MGUS patients, for whom clinical follow up data was available (*n* = 26; median follow-up: 284 weeks [range: 21–666 weeks]). β-gal staining conducted on MSCs from *n* = 24 MGUS patients revealed positivity ranging from 2.4–43.5% (median: 12.7%). Notably, MGUS patients who subsequently progressed to MM had a significantly higher percentage of β-gal-positive MSCs than those who had stable disease for 5 years or more (*p* = 0.0053, Kruskal-Wallis test with Dunn’s post-test; Supplementary Fig. 2). Furthermore, ROC curve analysis demonstrated that the level of β-gal staining could differentiate MGUS patients who subsequently progressed to MM from those with stable disease (AUC: 0.895; 95% CI: 0.75–1.00), with a cut-off of 10% β-gal-positive cells providing a sensitivity of 1.00 and a specificity of 0.59 and a cut-off of 27.5% β-gal-positive cells providing a sensitivity of 0.71 and a specificity of 1.00 (Fig. 2A). Strikingly, patients in the β-gal^hi^ group (≥27.5% β-gal-positive cells) were significantly more likely to progress to active MM than those in the β-gal^lo^ group (<10% β-gal-positive cells) (hazard ratio: 20.3 [95% CI: 2.2.5–162.9]; *p* = 0.0047, log-rank test; Fig. 2B). Notably, none of the 10 β-gal^lo^ patients progressed during follow up, while 2 of 8 patients in the β-gal^mid^ group, and 5 of 6 patients in the β-gal^hi^ group, progressed to active MM (median time-to-progression β-gal^mid^: 441.4 weeks, median time-to-progression β-gal^hi^: 243.9 weeks; Fig. 2B). This suggests that β-gal-positivity is a strong prognostic marker of risk of progression in MGUS patients.

To determine whether elevated MSC β-gal staining was associated with other clinical factors, we compared known risk factors of MGUS-to-MM progression (age, BM PC %, paraprotein type and paraprotein level, SFLC level and ratio, FISH/cytogenetic abnormalities) [3–5, 26–28] in the β-gal^lo^, β-gal^mid^ and β-gal^hi^ cohorts. Of clinical features that have previously been reported as being associated with a high-risk of MGUS to MM progression [3–5, 26–28], BM plasmacytosis was found to be significantly associated with β-gal positivity, with BM PC percentage being significantly elevated in the β-gal^hi^ group when compared with the β-gal^lo^ group (*p* = 0.016, Kruskal-Wallis with Dunn’s post-test) and BM PC ≥ 5% being observed in 5 of 6 (83%) patients in the β-gal^hi^ group compared with 1 of 10 (10%) in the β-gal^lo^ group (*p* = 0.008; Fisher’s exact test). This suggests a potential association between the level of MSC senescence and elevated PC burden in MGUS patients. Consistent with this, MSC β-gal staining significantly correlated with the bone marrow PC % in the MGUS cohort (*p* = 0.0002; Pearson correlation; R = 0.74; CI [0.43–0.89]; Fig. 2C), suggesting that these two variables are interrelated. However, the positive association between β-gal staining and MM PC % was not observed in the MM cohort (*p* = 0.15; Pearson correlation; R = -0.56; CI [–0.91 to –0.24]; Supplementary Fig. 3). Additionally, there was a significant difference in paraprotein isotype between groups (*p* = 0.04, β-gal^hi^ versus β-gal^lo^, Fisher’s exact test), with IgA predominating in the β-gal^hi^ group (17% IgG and 83% IgA) while IgG predominated in the β-gal^mid^ (70% IgG, 20% IgA, 10% light chain only) and β-gal^lo^ (88% IgG, 12% light chain only) groups (Table 1). There was a trend towards increased age in the β-gal^hi^ group, although this did not reach statistical significance (*p* = 0.10; Kruskal-Wallis test with Dunn’s post-test, Table 1).

**Table 1:** Clinical and senescent characteristics in β-gal^hi,^ β-gal^mid^ and β-gal^lo^ MGUS patients.

	β-gal^lo^	β-gal^mid^		β-gal^hi^	
	*n* = 10	*n* = 8	*P*^a^	*n* = 6	*P*^a^
**Age in years, median (range)**					
Median (range)	60 (42–78)	66 (59–83)	>0.99	75.5 (50–84)	0.10
Data missing (n)	0	0		0	
**Sex, no. (%)**			0.63		>0.99
Women	3 (30%)	4 (50%)		1 (17%)	
Men	7 (70%)	4 (50%)		5 (83%)	
**Clinical characteristics**					
BM PC %					
Median (range)	2.5 (1–6)	5 (2–8)	0.06	5.5 (5–9)	0.02
Data missing (n)	0	1		0	
Immunoglobulin isotype, no. (%)			0.73		0.04
IgG	7 (70%)	7 (88%)		1 (17%)	
IgA	2 (20%)	0 (0%)		5 (83%)	
Light chain only	1 (10%)	1 (12%)		0 (0%)	
Paraprotein, g/L					
Median (range)	8 (6–16)	12 (4–15)	>0.99	10 (3–28)	>0.99
Data missing or no paraprotein (n)	3	3		0	
SFLC (involved chain), mg/L					
Median (range)	13.5 (8–70)	414 (10–532)	0.15	205 (16–638)	0.20
Data missing (n)	4	3		2	
SFLC ratio (involved:uninvolved)					
Median (range)	1.5 (1.1–4)	25 (1.4–34.7)	0.14	10.8 (1.6–63.8)	0.36
Data missing (n)	4	3		2	
FISH/cytogenetics					
Abnormal FISH/cytogenetics, no. (%)	0 (0%)	0 (0%)	NA	0 (0%)	NA
Data missing (n)	5	7		7	
**High risk features**					
Age >65 years^d,e^, no. (%)	4 (40%)	4 (50%)	>0.99	5 (83%)	0.14
Age ≥60 years^f^, no. (%)	5 (50%)	8 (87.5%)	0.15	5 (83%)	0.31
BM PC ≥ 5%^f^, no. (%)	1 (10%)	5 (71%)	0.03	5 (83%)	0.008
Paraprotein ≥15 g/L^b,c,d,f^, no. (%)	1 (14%)	2 (40%)	0.53	2 (33%)	0.56
Non-IgG MGUS^b,c^, no. (%)	3 (30%)	1 (12%)	0.59	5 (83%)	0.12
Involved SFLC level (>100 mg/L)^d,e^, no. (%)	0 (0%)	3 (60%)	0.13	2 (50%)	0.06
Abnormal SFLC ratio (>1.65)^b,c,d,f^, no. (%)	2 (33%)	4 (80%)	0.24	3 (75%)	0.52

*BM PC* bone marrow plasma cell, *FISH* fluorescence in situ hybridisation, *Ig* immunoglobulin, *MGUS* monoclonal gammopathy of undetermined signficance, *MSC* mesenchymal stromal cell, *SFLC* serum free light chain.

^a^*p* values, compared to the β-gal^lo^ group, were calculated using Kruskall-Wallis test with Dunn’s post-test or Fisher’s exact test, where appropriate.

^b^Rajkumar et al. Blood 2005.

^c^Turesson et al. Blood 2014.

^d^Gran et al. Am J Hematol 2021.

^e^Cesana et al. J Clin Oncol 2002.

^f^Kyle et al. NEJM 2018.

β-gal^lo^: MSC β-gal % <10%; β-gal^mid^: MSC β-gal % 10-27.5%; β-gal^hi^: MSC β-gal % ≥27.5%.

In univariate analyses, high MSC β-gal staining (β-gal^hi^ ≥ 27.5%) was significantly associated with increased risk of progression to MM, when compared with the β-gal^mid/lo^ group (*p* = 0.025, Cox proportional hazards test, Table 2). Additionally, BM PC burden ≥5% was predictive of risk of progression (*p* = 0.083) in our cohort. In contrast, risk factors including age ≥60 years (*p* = 0.47), serum paraprotein levels ≥15 g/L (*p* = 0.45), non IgG paraprotein type (*p* = 0.55), SFLC level >100 mg/L (*p* = 0.18) and SFLC ratio >1.65 (*p* = 0.32) were not predictive of risk of progression. When β-gal staining and PC burden were included in multivariable analyses, β-gal^hi^ retained its significant predictive power (*p* = 0.042). Collectively, these data suggest that MSC β-gal positivity is an independent predictor of MM progression risk in MGUS patients.

**Table 2:** Univariate and multivariable Cox proportional hazards analysis.

Covariate	Univariate			Multivariable		
	OR	95% CI	*P*	OR	95% CI	*P*
β-gal^hi^ MSC ≥ 27.5%	6.99	1.27–39.4	0.025	16.2	1.10–238	0.042
Age >65 years	0.889	0.198–3.99	0.88			
Age ≥60 years	0.533	0.096–2.98	0.47			
BM PC ≥ 5%	6.53	0.785–54.3	0.083	0.817	0.053–12.6	0.89
Paraprotein ≥15 g/L	1.93	0.348–10.7	0.45			
Non-IgG MGUS	1.53	0.376–6.195	0.55			
Involved SFLC level >100 mg/L	4.79	0.496–46.3	0.18			
Abnormal SFLC ratio (>1.65)	60.6	0.019–200,000	0.32			

*β-gal* β-galactosidase, *BM PC* bone marrow plasma cell, *CI* confidence interval; *Ig* immunoglobulin, *MGUS* monoclonal gammopathy of undetermined signficance, *MSC* mesenchymal stromal cell, *OR* overall risk, *SFLC* serum free light chain.

### The capacity of MSCs to suppress MM cell growth in vitro inversely correlates with the level of MSC senescence

**Fig. 3:**
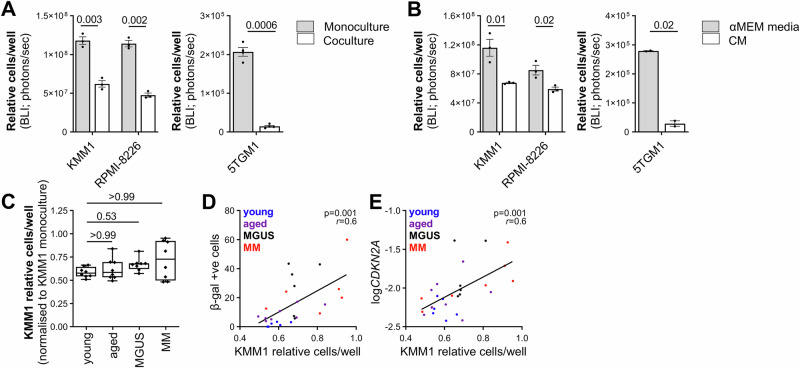
In vitro growth of MM cells is suppressed by MSCs, but suppression inversely correlates with the level of MSC senescence. **A** Luciferase-expressing MM cell lines were cultured alone (monoculture) or in coculture with MSCs from young non-cancer controls (KMM1, RPMI-8226) or eight-week-old mice (5TGM1). After 3 days, the relative number of MM cells was quantified by bioluminescence imaging (BLI). Graphs depict mean ± SEM of three (KMM1, RPMI-8226) or four independent experiments (5TGM1). **B** Luciferase-expressing MM cell lines were cultured in conditioned media (CM) from MSCs from young non-cancer controls (KMM1, RPMI-8226) or eight-week-old mice (5TGM1) or non-conditioned medium (αMEM media). After 3 days, the relative number of MM cells was quantified by BLI. Graphs depict mean ± SEM of three independent experiments (KMM1, RPMI-8226) or mean ± range of two independent experiments (5TGM1). **C** MSCs from young (*n* = 8) or aged (*n* = 8) non-cancer controls or MGUS (*n* = 8) or MM (*n* = 8) patients were seeded 24 hours prior to adding luciferase-expressing KMM1 cells. After 3 days, relative KMM1 cell numbers per well were quantitated using BLI and normalised to KMM1 cell numbers in monoculture. Box and whisker plots depict median and interquartile ranges. Scatter dot plots showing correlation of MSC % β-gal positivity (**D**) and *CDKN2A* expression (**E**) with relative KMM1 cell number in coculture with MSCs from young and aged non-cancer controls and MGUS and MM patients, normalised to cell numbers in monoculture. *p* values are shown for paired*t* tests (**A**, **B**), Kruskal-Wallis test with Dunn’s post-test (**C**) or Pearson’s correlation (**D**, **E**).

Next, we assessed the effects of MSCs from MGUS or MM patients or human and mouse non-cancer controls on growth of MM cell lines in coculture. Notably, coculture with human MSCs from healthy young controls for 3 days led to significantly lower MM cell numbers per well (as assessed by bioluminescence imaging), when compared with MM cells cultured alone, in the human MM cell lines KMM1 and RPMI-8226. Similarly, coculture with MSCs from eight-week-old syngeneic C57BL/KaLwRij mice for 3 days led to a significantly lower number of mouse 5TGM1 MM cells per well, when compared with 5TGM1 cells cultured alone (Fig. 3A). Suppression of MM cell numbers was also observed with conditioned media from healthy young human and mouse MSCs, when compared with complete αMEM media (Fig. 3B). In contrast, MSCs from 3/8 MM donors tested had minimal effects on KMM1 numbers after 3 days, suppressing KMM1 numbers by less than 10%, when compared with KMM1 cell numbers in monoculture (Fig. 3C). However, there was no statistically significant difference in the capacity of MGUS or MM MSCs to affect relative KMM1 cell numbers in coculture, compared with that of younger or older, non-cancer controls (*p* = 0.31, Kruskal-Wallis test; Fig. 3C). Strikingly, overall, we found a strong inverse correlation between the suppressive effect of MSCs on KMM1 cell proliferation and the level of MSC senescence, with relative KMM1 numbers in coculture positively correlating with MSC β-gal positivity (*p* = 0.001; Pearson’s *r* = 0.6 [95% CI: 0.3 to 0.8]; Fig. 3D) and *CDKN2A* expression (*p* = 0.001; Pearson’s *r* = 0.6 [95% CI: 0.3–0.9]; Fig. 3E). Additionally, Kaplan-Meier analysis revealed that MGUS patients with MSCs that had a greater suppressive effect on KMM1 cell number (<69% of the numbers observed in KMM1 monoculture; KMM1-prolif^lo^) were more likely to have stable disease, compared with patients whose MSCs had less of a suppressive effect on KMM1 cell numbers ( ≥ 69% of KMM1 in monoculture; KMM1-prolif^hi^) (*p* = 0.0013; log-rank test; Supplementary Fig. 4).

### Senescent MSCs support the growth of MM cells in vitro

**Fig. 4:**
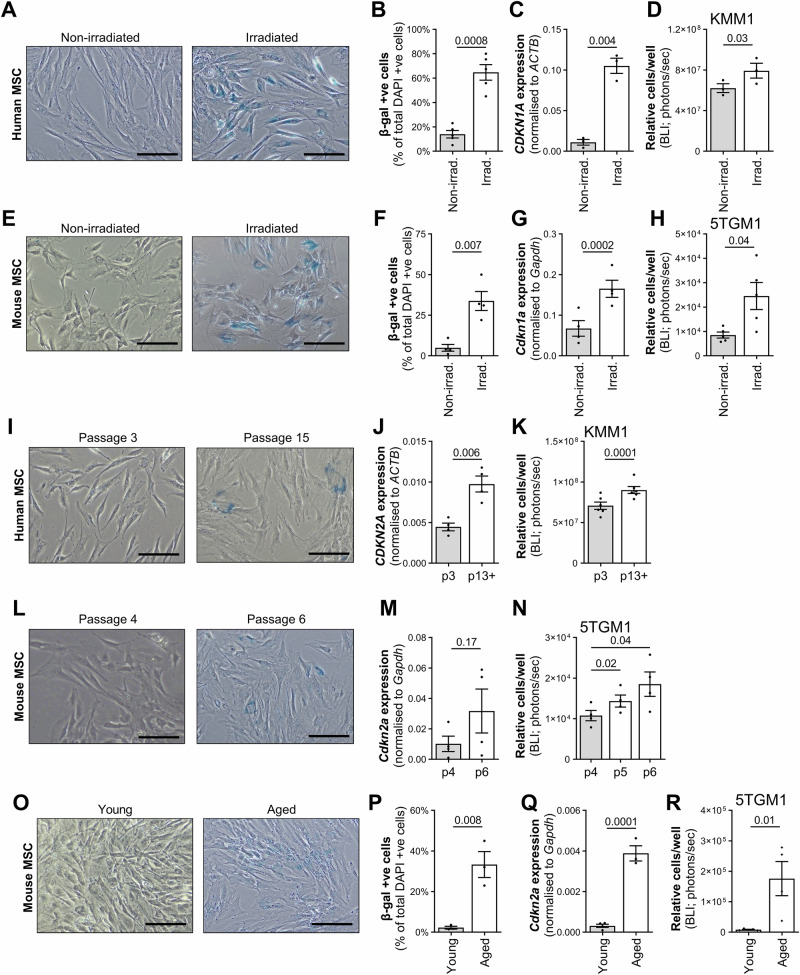
Irradiation-, replication- and ageing-induced MSC senescence alleviates MSC-induced suppression of MM cell growth in vitro*.* **A** Human MSCs from young non-cancer controls were irradiated at 60 Gy and β-gal activity (blue) was evaluated after 10 days and compared with non-irradiated cells. **B** β-gal-positive cells were quantitated in irradiated and non-irradiated cultures relative to total cell number (identified by DAPI co-stain). **C** Expression of *CDKN1A* was assessed in irradiated and non-irradiated MSCs and normalised to *ACTB*. **D** Irradiated MSCs (day 7) from young non-cancer controls and donor-matched non-irradiated controls were cocultured with luciferase expressing KMM1 cells. After 3 days, the relative number of KMM1 cells was enumerated using BLI. **E** MSCs from eight-week-old C57BL/KaLwRij mice were irradiated at 60 Gy and β-gal activity (blue) was evaluated after 4 days and compared with donor-matched non-irradiated cells. **F** β-gal-positive cells were quantitated relative to total DAPI-positive cell number. **G** Expression of *Cdkn1a* was assessed, normalised to *Gapdh*. **H** Irradiated and non-irradiated C57BL/KaLwRij MSCs were seeded and, after adhering overnight, coculture was initiated with luciferase-expressing 5TGM1 cells. After 3 days, the relative number of 5TGM1 cells was enumerated by BLI. **I** Human MSCs cultures, isolated from young non-cancer controls were passaged twice weekly until they reached replicative senescence (passage 13–15), as demonstrated by assessment of β-gal activity. **J** Expression of *CDKN2A*, normalised to *ACTB*, was analysed at passage 3 and at the passage of senescence (passage 13–15) in MSCs from young non-cancer controls. **K** MSCs from young non-cancer controls were cocultured with luciferase-expressing KMM1 cells for 3 days and the relative number of KMM1 cells was enumerated using BLI. **L** β-gal activity was assessed in MSC cultures from eight-week-old C57BL/KaLwRij mice at passage 4 and passage 6. **M** Expression of *Cdkn2a*, normalised to *Gapdh*, was analysed in C57BL/KaLwRij mouse MSCs. **N** Luciferase-expressing 5TGM1 cells were cocultured with mouse MSCs at passage 4, 5 and 6 and, after 3 days, 5TGM1 cells were enumerated using BLI. **O** MSCs were isolated by plastic adherence from long bones from young (eight-week-old) and aged (18-month-old) C57BL/KaLwRij mice, cultured for two weeks in vitro and cells were stained for β-gal activity at passage 3. **P** The percentage of β-gal-positive cells, was calculated relative to DAPI-positive cell number. **Q** Expression of *Cdkn2a*, normalised to *Gapdh*, was analysed in mouse MSCs from young and aged donors (*n* = 3–4 donors/group). **R** Mouse MSCs from young and aged donors were cocultured with luciferase-expressing 5TGM1 MM PCs for 3 days and 5TGM1 cells were quantitated by BLI. Representative images of β-gal-stained cells are shown (**A**, **E**, **I**, **L**, **O**). Scale bar: 200 µm. Graphs depict mean ± SEM of three (**C**, **D**, **G**, **P**–**R**), four (**F**, **H**, **J**, **M**, **N**, **Q**), five (**B**) or six (**K**) independent donors. *p* values are shown for paired *t* test (**B**–**D,** **F**-**H,** **J**, **K**, **M**), one-way ANOVA with Holm-Šídák’s post-test (**N**) or unpaired *t* test (**P**–**R**).

To investigate the effect of BM MSC senescence on MM cell growth, we used gamma irradiation to induce senescence in MSCs from healthy young controls, as confirmed by increased β-gal staining (Fig. 4A, B) and expression of irradiation-induced senescence marker *CDKN1A* (Fig. 4C). Coculture with irradiated senescent MSCs for 3 days increased human KMM1 MM cell numbers by 30%, in comparison to coculture with non-irradiated control MSCs (*p* = 0.03, paired *t* test; Fig. 4D). Similarly, coculture with irradiation-induced senescent primary mouse MSCs (Fig. 4E–G) increased 5TGM1 cell numbers three-fold relative to coculture with non-irradiated control mouse MSCs (*p* = 0.03, paired *t* test; Fig. 4H).

Additionally, we induced replicative senescence in MSCs from healthy young controls by extended culture to passage 13–15 with senescence being confirmed by increased β-gal staining and *CDKN2A* expression (Fig. 4I, J). Replication-induced senescent (passage 13–15) human MSCs significantly increased KMM1 cell numbers, when compared with early passage (passage 3) MSCs (*p* = 0.03, paired *t* test; Fig. 4J). Similar results were observed with sequential passage of MSCs from MGUS and MM donors (Supplementary Fig. 5) and with replication-induced senescent mouse MSCs (Fig. 4L–N).

Finally, we assessed the ability of MSCs from aged 18-month-old mice, compared with MSCs from young 2-month-old mice, to support the growth of 5TGM1 cells in vitro. MSCs from aged mice were significantly more senescent, as reflected by β-gal positivity (Fig. 4O, P) and *Cdkn2a* expression (Fig. 4Q), and had a significant, 20-fold increased capacity to support 5TGM1 growth in coculture, when compared with those of young donor animals (p = 0.011, unpaired *t* test; Fig. 4R).

Collectively, these data show that while non-senescent MSCs suppress MM cell numbers in coculture, this effect is relieved by induction of senescence in MSCs, enabling the growth of MM cell lines in vitro.

### Gremlin1 is a novel SASP factor that may enable the growth of MM cells in coculture with senescent MSCs

**Fig. 5:**
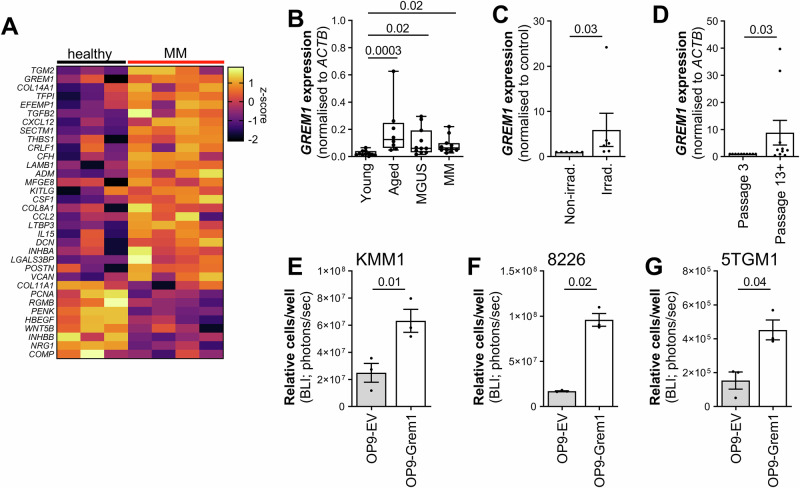
Identification of Gremlin1 as a pro-myeloma SASP factor that is expressed by MGUS and MM MSCs. **A** In silico analysis of gene expression in BM MSCs from MM patients (*n* = 4) and age-matched healthy controls (*n* = 3) in publicly available microarray dataset GSE36474. Heat map shows the average z-score for significantly up- or down-regulated genes. **B** Expression of *GREM1*, normalised to *ACTB*, was analysed in passage 5 MSCs from young non-cancer controls (*n* = 11), aged non-cancer controls (*n* = 8) and newly diagnosed MGUS (*n* = 11) and MM (*n* = 12) patients. Box and whisker plots depict median and interquartile ranges. **C** Human MSCs from young non-cancer controls (*n* = 6) were irradiated (60 Gy) to induce senescence and *GREM1* expression, normalised to *ACTB*, was assessed. Data are normalised to non-irradiated cells. **D** Replicative senescence was induced in human MSCs from young non-cancer controls (*n* = 11) by sequential passage (passage 13–15) and *GREM1* expression, normalised to *ACTB*, was analysed. Data are normalised to low passage (passage 3) cells. Luciferase-expressing KMM1 (**E**), RPMI-8226 (**F**) and 5TGM1 (**G**) cells were cocultured with OP9 cells overexpressing Gremlin1 (OP9-Grem1), and empty vector control cells (OP9-EV) and, after three days, the relative number of MM cells was quantitated using BLI (*n* = 3 independent experiments). Graphs depict mean ± SEM. *p* values are shown for Kruskal-Wallis test with Dunn’s post-test (**B**) or Wilcoxon matched-pairs signed rank test (**C**, **D**) or paired *t* tests (**E–G**).

Next, we identified potential factors secreted by senescent MSCs (SASP factors) that may affect MM cell growth. In order to identify potential SASP factors produced by MSCs in MM patients, we analysed publicly available microarray data from MSC cultures from MM patients (*n* = 4) and healthy controls (*n* = 3). The mRNA expression of 25 genes encoding secreted or cell surface ligands were significantly upregulated, and 9 were significantly downregulated, in MM MSCs when compared with healthy control MSCs (limma, FDR *p* value < 0.05; Fig. 5A). Notably, one of the most highly upregulated secreted factor genes was *GREM1* (encoding the BMP2/4/7 antagonist Gremlin1) which we have previously shown to play a role in MM tumour development in vivo [23].

In order to determine if Gremlin1 is upregulated in senescent MSCs, we assessed *GREM1* expression in BM from young and aged donors, by qRT-PCR. *GREM1* was significantly upregulated in MSCs from older non-cancer controls compared with young non-cancer controls (*p* = 0.0003; Kruskal Wallis test with Dunn’s multiple comparisons post-test; Fig. 5B). Furthermore, *GREM1* expression was upregulated in MSCs from individuals with MGUS and MM, when compared with healthy young donors (*p* = 0.018 and *p* = 0.021, respectively; Kruskal Wallis test with Dunn’s multiple comparisons post-test; Fig. 5B). Similarly, *GREM1* was found to be upregulated in MM MSCs, when compared with control MSCs, in 3 independent transcriptomic datasets (Supplementary Fig. 6). Additionally, we found that *GREM1* expression was significantly upregulated following irradiation (*p* = 0.031; Wilcoxon signed rank test; Fig. 5C) or replicative exhaustion (*p* = 0.021; Wilcoxon signed rank test; Fig. 5D) in MSCs from young non-cancer controls. Taken together, these data suggest that Gremlin1 may be a novel SASP factor.

We have previously shown that MSC-derived Gremlin1 can stimulate the proliferation of 5TGM1 cells in vitro [23]. Here, OP9 stromal cells transduced to over-express Gremlin1, or empty vector control OP9 cells, were cocultured with KMM1, RPMI-8226 or 5TGM1 MM cell lines. KMM1, RPMI-8228 or 5TGM1 cell numbers were increased more than 2.5-fold in coculture with Gremlin1 over-expressing OP9 cells compared with empty vector OP9 cells (*p* = 0.010, *p* = 0.031 and *p* = 0.036, respectively; paired *t* test, Fig. 5E–G) after 72 h. These data demonstrate that Gremlin1 expression in MSCs increases the growth of both human and murine MM cells.

## Discussion

Advancing age is a major risk factor for the development of many cancers [8, 29]. The accumulation of senescent cells in the tumour microenvironment with ageing has been implicated in promoting malignant cell outgrowth in solid cancers [7, 30–37] as well as haematological malignancies such as acute myeloid leukaemia [8, 38]. In MM, it has been reported that MSCs isolated from MM patients display senescent characteristics, including elevated p16 expression and reduced proliferation [11–15, 18]. Here, we have shown for the first time that elevated MSC senescence is a feature of patients with MGUS. Moreover, there is a strong association between MSC senescence in MGUS patients and risk of subsequent progression to active MM, suggesting that the accumulation of senescent MSCs may be a driver of this process. Importantly, we also showed that features of senescence including β-gal positivity, *CDKN2A* expression and proliferative rate in MSCs from MGUS and MM patients and from non-cancer controls strongly correlated with donor age. Taken together, this indicates that MSC senescence in MGUS and MM patients is, at least in part, reflective of ageing-related senescence.

Notably, our studies showed that MSCs from healthy, non-cancer controls suppress the growth of MM cell lines. However, our observation that the level of MSC senescence positively correlates with KMM1 cell numbers suggests that the suppressive effect is relieved by MSC senescence. These findings are in line with previous studies demonstrating MM cell line proliferation is decreased in coculture with MSCs from healthy donors and increased in coculture with MSCs from MM patients [10, 11, 39–41]. Our results are further supported by previous studies whereby Dicer1 knockdown-mediated senescence induction in MSCs from healthy donors significantly stimulated the proliferation of the MM cell line NCI-H929 in coculture assays, compared with control MSCs [13]. Notably, we found that reduced in vitro KMM1 cell growth-suppressive capacity of MSCs from MGUS patients and the level of senescence in these MSCs was positively associated with the risk of progression to MM in these patients. Taken together, these findings suggest a hypothesis whereby increased MSC senescence in the BM related to ageing could increase the proliferation of clonal PCs, leading to progression from MGUS to MM.

The potential direct association between MSC senescence and MM development is further supported by our data demonstrating that BM PC burden was significantly higher in the β-gal^hi^ MGUS patient cohort. This may reflect early proliferative changes in clonal PCs that are driven by senescent MSCs, prior to progression to MM. However, it is also plausible that this association may be influenced by tumour cell-mediated induction of MSC senescence, with some studies reporting that coculture of primary MM cells, or MM cell lines, with healthy human MSCs induces a senescent phenotype in MSCs, compared with MSCs in monoculture [13, 18, 42]. MM cells may therefore, under some conditions, increase MSC senescence to exacerbate the senescent phenotype. While patient age and BM PC burden are potential drivers of MSC senescence in MGUS patients, our data also suggest a potential association between MSC senescence and other high-risk clinical features, such as non-IgG immunoglobulin subtype. However, a direct causative association between immunoglobulin type and MSC senescence remains to be confirmed.

While the underlying mechanisms by which senescent MSCs support MM cell growth is currently unclear, it is possible that SASP factors may play a role. Here, we have identified Gremlin1 as a potential novel SASP factor in BM MSCs, with *GREM1* expression being significantly elevated in human MSCs with ageing or following irradiation- or replicative exhaustion-induced senescence. This is consistent with previous proteomic analysis that identified Gremlin1 as a component of the SASP in BM MSCs and lung fibroblasts following senescence induction [43, 44]. Gremlin1 is a secreted factor which is a member of the DAN family of bone morphogenetic protein (BMP) antagonists, expressed by MSC-lineage cells in a range of tissues, which directly binds to BMP2, BMP4 and BMP7 and prevents them from binding to, and signalling via, their cognate receptors [45, 46]. BMP2, BMP4 and BMP7 are expressed by mesenchymal lineage cells in the BM microenvironment and have been consistently shown to decrease cell proliferation and, in some cases, induce apoptosis in human MM cell lines [47–52]. Our finding that *GREM1* expression in MSCs is significantly upregulated with ageing and senescence induction suggests that increased Gremlin1 expression, as part of the SASP, may play a critical role in counteracting the growth suppressive effects of BMPs, thereby facilitating the proliferation of MM cells. Indeed, we found that the growth of human MM cells KMM1 and RPMI-8226 and the mouse MM cell line 5TGM1 were significantly increased in coculture with mouse mesenchymal OP9 cells overexpressing Gremlin1, compared with empty vector control OP9 cells. This is consistent with our previous studies demonstrating that supplementation of anti-Gremlin1 neutralising antibody to 5TGM1 and OP9 stromal cell cocultures reduced 5TGM1 proliferation in vitro, when compared with isotype-control antibody [23]. Furthermore, treatment of 5TGM1 tumour-bearing C57BL/KaLwRij mice with an anti-Gremlin1 neutralising antibody significantly decreased tumour burden in the BM, when compared with isotype controls [23]. Notably, elevated Gremlin1 expression in the tumour stromal microenvironment has been linked to faster progression in colorectal, breast, cervical and pancreatic cancer [53]. Whether Gremlin1 could be utilised as a biomarker to predict MGUS patients at risk of progression to MM warrants further investigation.

In addition to Gremlin1, other SASP factors may contribute to the pro-myeloma effect of senescent MSCs. MSCs from MM patients have previously been found to display an altered secretory profile compared with that of MSCs from age-matched non-cancer controls, closely resembling the SASP of senescent human MSCs [13, 14, 54]. Consistent with these studies, our in silico analysis revealed MM MSCs express several known SASP factors, including *EFEMP1*, *THBS1*, *CFH*, *LGALS3BP* and *POSTN* [43, 44]. In addition, the well-established canonical SASP factor and pro-inflammatory cytokine IL-6 has previously been shown to be upregulated in MM MSC [11, 13, 14, 17, 55, 56]. and is well-documented as a critical factor for the survival and proliferation of MM tumour cells [11, 57–61]. Notably, MSC-derived IL-6 has been shown to promote MM cell growth and survival, with the addition of an anti-IL-6 monoclonal antibody abrogating the proliferation of MM cell lines in coculture with MSCs [11, 61]. As it is well established that IL-6 secretion is upregulated in senescent MSCs [43, 62], MSC-derived IL-6 may play a key role in the favourable BM microenvironment created by senescent MSCs for MM PC growth.

In summary, our findings have identified, for the first time, that MSC senescence is evident in MGUS patients and may play important biologic and prognostic roles in the progression from MGUS to overt MM disease. Importantly, we have shown that MSC senescence alleviates the suppressive effects of MSCs on growth of MM cell lines in coculture, suggesting that senescent MSCs create a favourable environment that may promote disease progression. Moreover, we propose that factors secreted by senescent MSCs as part of the SASP, including Gremlin1, may play an integral role in counteracting the growth suppressive effects of healthy MSCs, thereby enabling MM PC outgrowth. Future studies are warranted to investigate whether targeted interventions that modulate senescent MSCs could disrupt their capacity to support MM cell growth. This may offer opportunities for therapeutic strategies to prevent or delay MGUS progression to overt MM disease.

## Supplementary information

Supplementary information

## Data Availability

The datasets generated during the current study are available from the corresponding author on reasonable request.
