# Supplementary material for: Age-related mesenchymal stromal cell senescence is associated with progression from MGUS to multiple myeloma
Source: Leukemia. 2025 Apr 22;39(6):1464–75. doi: 10.1038/s41375-025-02621-7 (PMC12133572; doi:10.1038/s41375-025-02621-7)
Supplement: Supplementary file 1 — Supplementary information [file 41375_2025_2621_MOESM1_ESM.pdf]

Title: Age-related mesenchymal stromal cell senescence is associated with progression from MGUS to multiple myeloma

Authors' names: Natalya Plakhova[1,2], Vasilios Panagopoulos[1,2], Melissa D. Cantley[1,2], Laura J. Trainor[1,2], Duncan R. Hewett[1,2], Kimberley C. Clark[1,2], Jo Gardiner[3], Angelina Yong[3], Cindy Lee[3], Noemi Horvath[3], Peter I. Croucher[4], Dimitrios Cakouros[2,5], Sheila A. Stewart[6], Stan Gronthos[2,5], Andrew C. W. Zannettino[1,2], Krzysztof M. Mrozik[1,2]*, Kate Vandyke[1,2]*

* These authors contributed equally to this work

Affiliations:

1. Myeloma Research Laboratory, School of Biomedicine, Faculty of Health and Medical Sciences, The University of Adelaide, Adelaide, Australia
2. Precision Cancer Medicine Theme, South Australian Health & Medical Research Institute (SAHMRI), Adelaide, Australia
3. Department of Clinical Haematology, Royal Adelaide Hospital, Adelaide, Australia
4. Garvan Institute of Medical Research, Sydney, Australia
5. Mesenchymal Stem Cell Laboratory, School of Biomedicine, Faculty of Health and Medical Sciences, The University of Adelaide, Adelaide, Australia
6. Department of Cell Biology and Physiology, ICCE Institute, Washington University School of Medicine, St. Louis, USA

Materials and Methods

Cell culture reagents

All cell lines and primary cells were maintained in a humidified 5% CO_2 incubator maintained at 37°C. Unless otherwise specified, cell culture reagents and consumables were sourced from Sigma-Aldrich. All media were supplemented with 2 mM L-glutamine, 100 U/mL penicillin, 100 µg/mL streptomycin, 1 mM sodium pyruvate and 10 mM HEPES buffer. Adherent cells were sub-cultured by incubation in 1 mg/mL collagenase type II (Worthington) and 1 mg/mL dispase II (Roche) in Hank's buffered salt solution (HBSS) for 30 minutes (murine MSCs) or 1 hour (human MSCs) followed by 0.05% (v/v) trypsin-EDTA for 1-5 minutes. Prior to use, all cell lines were tested for mycoplasma infection using a MycoAlert Mycoplasma Detection Kit (Lonza) following the manufacturer's instructions.

Primary human MSC cultures

MSCs from healthy young volunteers (*n*=14; 8 male and 6 female; aged 17-32 years) were obtained from trabecular bone fragments from posterior superior iliac crest BM aspirates. All individuals were screened for common human viral pathogens and a review of their blood and BM was performed by an experienced pathologist to confirm they were haematologically normal prior to culture of their MSCs. MSCs from aged donors without any concomitant cancer diagnosis (non-cancer individuals) were kindly provided by Prof. Gerald Atkins (The University of Adelaide), isolated from intertrochanteric trabecular bone samples from patients undergoing hip arthroplasty for neck of femur fractures (*n*=8; 4 male and 4 female; aged 65-94 years). MSCs from patients with MGUS (*n*=30; 19 male and 11 female; aged 42-84 years) or newly diagnosed, previously untreated symptomatic MM (*n*=13; 9 male and 4 female; aged 52-86 years) were isolated from trabecular bone from iliac crest biopsies through the South Australian Cancer Research Biobank (SACRB).

Primary MSC cultures were obtained by plastic adherence as previously described[1, 2] and were cultured in α-modified minimum essential medium (αMEM) with 20% foetal bovine serum (FBS; Thermo Fisher Scientific) and 100 µM L-ascorbate-2-phosphate and supplements (complete αMEM). Cells were expanded to passage 3 and were then sub-cultured twice per week (every 3-4 days). MSC cultures were confirmed to fulfil the minimal criteria for defining human MSC[3] (Supplementary Figure 1). Briefly, flow cytometry was used to confirm expression of CD105, CD90, CD73 and negativity for CD45, CD34 and CD14 (as determined by flow cytometry, as described previously[4]). Furthermore, the capacity of the MSC cultures

to form adipocytes and undergo mineralization *in vitro* was confirmed using established protocols.[1, 5]

In order to determine the proliferative capacity of human MSCs, from passage 3 onwards, MSCs were seeded at 6×10^3 cells/cm^2 in triplicate 25 cm^2 flasks in complete αMEM, and cultured for 3 days. MSCs were then harvested and viable cells were counted using trypan blue dye exclusion.

At passage 5, cells were seeded at 2.5×10^4 cells/cm^2 in 96-well black-walled flat bottom plates (Corning Life Science) for coculture with MM PCs or were seeded at 1×10^4 cells/cm^2 in 6-well plates for assessment of β-gal activity and RNA isolation for qRT-PCR, as described below.

For the MGUS patients, initial passage to senescence analysis was conducted on n=10 donors. Analysis of proliferative rate, β-gal staining and qRT-PCR analysis of senescence markers was performed on n=9 MGUS patients at passage 5. Coculture with human MM cell line KMM1 was performed in n=8 of 9 of these donors. For *CDKN2A* (Fig 1F) and *GREM1* qRT-PCR analyses, an additional n=2 donors were included to achieve sufficient statistical power (total: n=11 donors). In order to analyse the association between the senescent phenotype and clinical features in MGUS patients, β-gal staining and coculture with KMM1 cells was performed on an additional cohort of n=15 MGUS patients (total patients included: n=24 for β-gal analysis, n=16 for KMM1 coculture analysis; Fig 2).

For the MM patients, analyses were conducted using a total of n=13 patients. Initial passage to senescence analysis was conducted on n=9 of 13 patients. Analysis of β-gal staining was performed on n=11 of 13 patients, analysis of MSC proliferative rate and coculture with KMM1 cells was conducted with n=8 of 13 patients, and qRT-PCR analysis of *CDKN2A* and *GREM1* expression was conducted on n=12 and n=11, respectively.

For the young non-cancer controls, analysis of passage to senescence, proliferative rate, β-gal staining and coculture with KMM1 MM cells was performed using a total of n=10 donors, with β-gal staining, proliferative rate analysis and coculture with KMM1 cells performed on n=8 of 10 donors. *CDKN2A* and *GREM1* gene expression data was only available for n=6 of these donors; as such, an additional cohort of n=5 donors, all at p5, was also included in this analysis, to ensure sufficient statistical power (total: n=11 donors).

For the aged non-cancer controls, insufficient cells were available to conduct passage to senescence analysis on all donors, so this data was not graphed for these patients. Analysis of MSC proliferative rate, β-gal staining, coculture with KMM1 MM cells and *CDKN2A* and *GREM1* gene expression was performed on n=8 donors.

Mouse MSC cultures

Primary mouse MSCs were isolated from the tibiae and femora from eight-week-old and 18-month-old C57BL/KaLwRij.Hsd mice, as approved by the SAHMRI animal ethics committee (SAM448.19). Bones were cleaned of muscle and connective tissue and were finely cut into small fragments and resulting bone chips were cultured in complete αMEM. Adherent cells were passaged at least twice before use in experiments.

The mouse MSC line OP9, originally obtained from the American Type Culture Collection (ATCC) was maintained in Dulbecco's modified Eagle's medium (DMEM) with 10% FBS and supplements (complete DMEM). Gremlin1 over-expressing and empty vector (EV) OP9 cell lines were previously generated using LEGOiT2 lentiviral vector (Plasmid #27343, Addgene) harbouring the murine cDNA for the coding region of *Grem1*, or vector alone, as previously described.[6]

Myeloma cell lines

The murine MM 5TGM1 cell line was originally kindly provided by Assoc. Prof. Claire Edwards (University of Oxford). The generation of a bone-tropic 5TGM1 subline engineered to express green fluorescent protein (GFP) and luciferase (5TGM1-luc) has previously been described.[7, 8] 5TGM1-luc cells were maintained in Iscove's modified Dulbecco's medium (IMDM) with 20% FBS and supplements (complete IMDM).

RPMI-8226 cells were obtained from the American Type Culture Collection. KMM1 cells were kindly gifted by Prof. Andrew Spencer (Monash University). KMM1 and RPMI-8226 cells were maintained in Roswell Park Memorial Institute 1640 (RPMI-1640) medium with 10% FBS and supplements (complete RPMI). The identity of the RPMI-8226 cells and KMM1 cell lines was confirmed by STR analysis performed at the Australian Genomics Research Facility (AGRF). Luciferase-expressing RPMI-8226-luc and KMM1-luc cells were generated using the SFG-NES-TGL vector, as described previously.[9]

Senescence induction by irradiation

MSCs were resuspended at 1×10^5 cells/mL in complete αMEM and irradiated at 60 Gy (4.5 Gy/minute for 13.3 minutes) using an RS2000 Biological Irradiator (Rad Source) at 160 keV with a tube current of 25 mA. Irradiated cells (and non-irradiated controls from the same donors) were seeded at 2.5×10^4 cells/cm^2 in 6-well plates for analysis of β-gal activity or gene expression (after 4 days [mouse] or 10 days [human]) or in 96-well black-walled flat bottom plates for coculture with MM PCs.

Senescence-associated β-galactosidase (β-gal) staining

Staining for β-gal activity was performed using the Senescence β-galactosidase Staining Kit #9860 (Cell Signaling Technology) according to the manufacturer's protocol. Cells were counterstained with 1 µg/mL 4′,6-diamidino-2-phenylindole (DAPI) (Thermo Fisher) nuclear stain in Milli-Q water in the dark at room temperature for 10 minutes. Cells were imaged at 20x magnification using an IX53 Olympus inverted fluorescence microscope and the percentage of β-gal positive cells was calculated as a proportion of total DAPI-positive cells in 3 fields of view per well in triplicate wells.

Quantification of MM cell numbers using bioluminescence imaging

For coculture experiments, MSCs or OP9 cells were seeded at 2.5×10^4 cells/cm^2 in 96-well black-walled flat bottom plates and were allowed to adhere overnight. Media were aspirated and 1×10^4 cells/well KMM1-luc, RPMI-8226-luc or 5TGM1-luc cells were seeded in 100 µL of complete RPMI (human MM cells) or complete IMDM (mouse MM cells) with MSCs (MSC:MM cell ratio of 5:1) or alone (monoculture). After three days of coculture, the relative number of MM cells per well was quantitated using bioluminescence imaging as described previously.[10] Briefly, MM cells were incubated in 150 µg/mL D-luciferin (L-8220, BioSYNTH) in complete media in the dark at 37°C for 20 minutes prior to measurement of the bioluminescent signal using the IVIS Spectrum Bioluminescence Imaging System (Perkin Elmer). Where appropriate, bioluminescence in coculture was normalised to that of MM cells in monoculture.

For conditioned media experiments, MSCs from humans (three independent young non-cancer donors) or mice (two independent eight-week-old animals) were seeded at 2.5×10^4 cells/cm^2 in 6-well plates and allowed to adhere overnight, and media were then replaced with fresh complete αMEM. Conditioned media were collected after 72 hours, and KMM1-luc, RPMI-8226-luc or 5TGM1-luc cells were cultured at 1×10^4 cells/mL in conditioned media or complete αMEM. After three days, the relative number of MM cells/well was quantitated using bioluminescence imaging.

Quantitative reverse transcription polymerase chain reaction (qRT-PCR)

qRT-PCR was performed using RT2 SYBR® Green qPCR Mastermix (QIAGEN) on a CFX Connect Real-Time PCR Detection System (Bio-Rad). Primer sequences for mouse and human genes are listed in Supplementary Table I. Gene expression levels were calculated relative to *ACTB* (human) and *Gapdh* (mouse) using the $2^{-\Delta CT}$ method.[11]

Suppementary Table I qRT-PCR primers

Gene	Species	Forward	Reverse
CDKN2A ($p16^{INK4A}$)	human	5’-GAAGGTCCCTCAGACATCCCC-3’	5’-CCCTGTAGGACCTTCGGTGAC -3’
Cdkn2A ($p16^{INK4A}$)	mouse	5’-TCTGCTCAACTACGGTGCAG-3’	5’-ATCGCACGATGTCTTGATGT-3’
CDKN1A ($p21^{Cip1}$)	human	5’-ATTAGCAGCGGAACAAGGAG-3’	5’-CTGTGAAAGACACAGAACAG-3’
Cdkn1a ($p21^{Cip1}$)	mouse	5’-GAACATCTCAGGGCCGAAAAC-3’	5’-CTGCGCTTGGAGTGATAGAA-3’
GREM1	human	5’-AGGCCCAGCACAATGACTCAG-3’	5’-GTCTCGCTTCAGGTATTTGCG-3’
Grem1	mouse	5’-GCGCAAGTATCTGAAGCGAG-3’	5’-CGGTTGATGATAGTGCGGCT-3’
ACTB	human	5’-GATCATTGCTCCTCCTGAGC-3’	5’-GTCATAGTCCGCCTAGAAGCAT-3’
Gapdh	mouse	5’-AGGTCGGTGTGAACGGATTTG-3’	5’-TGTAGACCATGTAGTTGAGGTCA-3’

Microarray data

Gene expression was compared in MSCs from n=3 healthy controls and n=4 MM donor MSCs using the publicly available microarray dataset GSE36474.[12] Raw microarray data (CEL files) were obtained from the Gene Expression Omnibus (NCBI) and were normalised with the robust multi-array average (RMA) algorithm using the Bioconductor package affy, $\log_2$ transformed,

and significant differences between groups were identified using limma in R (version 3.03), as previously described.[8] Analysis was restricted to secreted and cell surface ligands, is defined in two independent databases (ConnectomeDB[13]; Human Protein Atlas[14]).

Power calculations

Power calculations were performed to determine the number of patients per group to include in characterization of the senescent phenotype in patient-derived MSCs. These analysis, based on pilot data, suggested that analysis of n=8 donors per group were required to detect significant difference in β-gal staining between MM or healthy controls and n=10 donors per group were required for qRT-PCR studies (statistical power = 0.80; α = 0.05).

References

1. Fitter S, Dewar AL, Kostakis P, To LB, Hughes TP, Roberts MM*, et al.* Long-term imatinib therapy promotes bone formation in CML patients. *Blood* 2008 Mar 1; **111**(5)**:** 2538-2547.

2. Stapledon CJM, Stamenkov R, Cappai R, Clark JM, Bourke A, Bogdan Solomon L, Atkins GJ. Relationships between the bone expression of Alzheimer's disease-related genes, bone remodelling genes and cortical bone structure in neck of femur fracture. *Calcif Tissue Int* 2021 May; **108**(5)**:** 610-621.

3. Dominici M, Le Blanc K, Mueller I, Slaper-Cortenbach I, Marini F, Krause D*, et al.* Minimal criteria for defining multipotent mesenchymal stromal cells. The International Society for Cellular Therapy position statement. *Cytotherapy* 2006; **8**(4)**:** 315-317.

4. Hynes K, Menicanin D, Mrozik K, Gronthos S, Bartold PM. Generation of functional mesenchymal stem cells from different induced pluripotent stem cell lines. *Stem Cells Dev* 2014 May 15; **23**(10)**:** 1084-1096.

5. Fitter S, Vandyke K, Gronthos S, Zannettino AC. Suppression of PDGF-induced PI3 kinase activity by imatinib promotes adipogenesis and adiponectin secretion. *J Mol Endocrinol* 2012 Jun; **48**(3)**:** 229-240.

6. Clark KC, Hewett DR, Panagopoulos V, Plakhova N, Opperman KS, Bradey AL*, et al.* Targeted disruption of bone marrow stromal cell-derived Gremlin1 limits multiple myeloma disease progression *in vivo*. *Cancers (Basel)* 2020 Aug 3; **12**(8).

7. Cheong CM, Chow AW, Fitter S, Hewett DR, Martin SK, Williams SA*, et al.* Tetraspanin 7 (TSPAN7) expression is upregulated in multiple myeloma patients and inhibits myeloma tumour development in vivo. *Exp Cell Res* 2015 Mar 1; **332**(1)**:** 24-38.

8. Noll JE, Vandyke K, Hewett DR, Mrozik KM, Bala RJ, Williams SA*, et al.* PTTG1 expression is associated with hyperproliferative disease and poor prognosis in multiple myeloma. *J Hematol Oncol* 2015 Oct 6; **8:** 106.

9. Diamond P, Labrinidis A, Martin SK, Farrugia AN, Gronthos S, To LB*, et al.* Targeted disruption of the CXCL12/CXCR4 axis inhibits osteolysis in a murine model of myeloma-associated bone loss. *J Bone Miner Res* 2009 Jul; **24**(7)**:** 1150-1161.

10. Mrozik KM, Cheong CM, Hewett D, Chow AW, Blaschuk OW, Zannettino AC, Vandyke K. Therapeutic targeting of N-cadherin is an effective treatment for multiple myeloma. *Br J Haematol* 2015 Jul 20.

11. Schmittgen TD, Livak KJ. Analyzing real-time PCR data by the comparative C(T) method. *Nature protocols* 2008; **3**(6)**:** 1101-1108.

12. Andre T, Meuleman N, Stamatopoulos B, De Bruyn C, Pieters K, Bron D, Lagneaux L. Evidences of early senescence in multiple myeloma bone marrow mesenchymal stromal cells. *PLoS One* 2013; **8**(3)**:** e59756.

13. Hou R, Denisenko E, Ong HT, Ramilowski JA, Forrest ARR. Predicting cell-to-cell communication networks using NATMI. *Nat Commun* 2020 Oct 6; **11**(1)**:** 5011.

14. Uhlen M, Karlsson MJ, Hober A, Svensson AS, Scheffel J, Kotol D*, et al.* The human secretome. *Sci Signal* 2019 Nov 26; **12**(609).

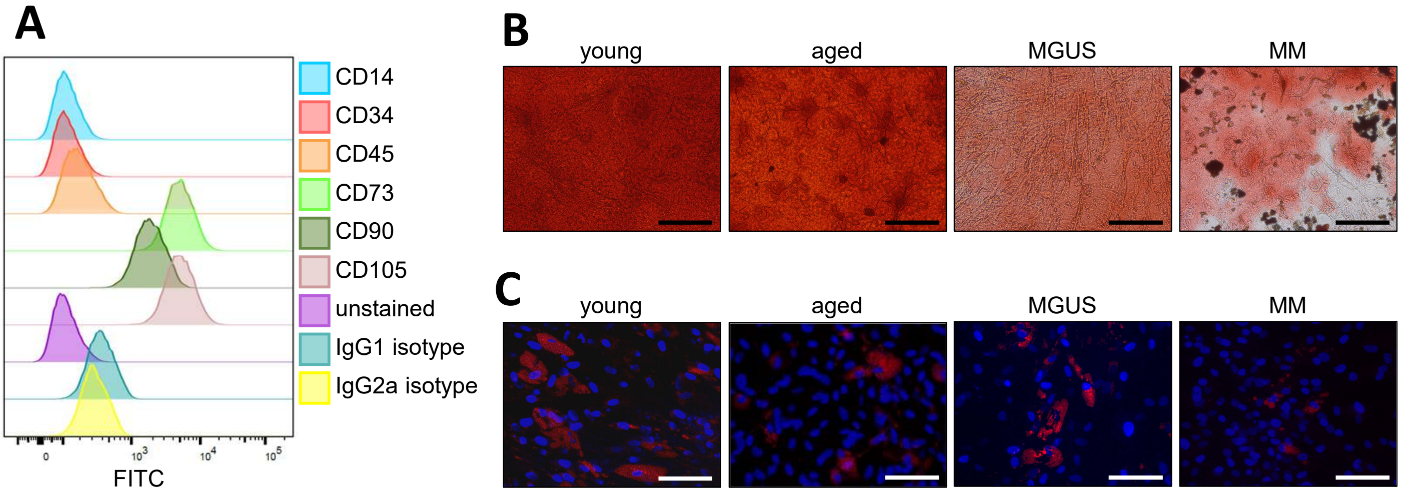

Supplementary Figure 1. Characterisation of MSC phenotype of MSC cultures from MGUS, MM and non-cancer individuals. A. Human MSCs were isolated by plastic adherence from bone chips obtained from the posterior iliac crest from MGUS and MM patients and healthy young volunteers, or from the proximal femur of aged, non-cancer controls individuals, and MSC phenotype was assessed at passage 4. Flow cytometric assessment of cell surface antigens is shown for a representative patient, revealing positivity for MSC markers CD73, CD90 and CD105 and negativity for CD14, CD34 and CD45. **B**. Mineral formation was assessed following culture of MSCs in αMEM with 5% FBS, 100 µM L-ascorbate-2-phosphate, 2.6 mM KH_2PO_4 and 100 nM dexamethasone for 21 days. Cells were then fixed in 10% formalin and mineral was stained with 2% alizarin red S. Images from representative donors are shown; scale bar: 100 µm. **C**. Adipogenic capacity was assessed following culture of MSCs in αMEM with 10% FBS, 100 µM L-ascorbate-2-phosphate, 60 µM indomethacin and 100 nM dexamethasone for 21 days. Cells were fixed in 10% formalin and stained with 25 ng/mL nile red and 300 nM DAPI to visualise lipids and nuclei, respectively. Images from representative donors are shown; scale bar: 25 µm.

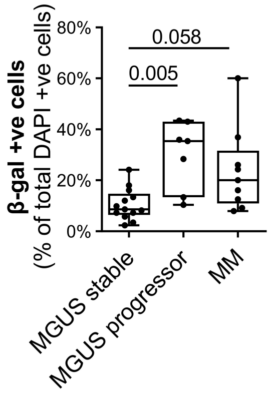

Supplementary Figure 2 MSC senescence is elevated in progressing MGUS patients. Percentage of β-gal-positive cells, as a proportion of total cells (identified by DAPI co-stain), was calculated for MGUS patients who subsequently progressed to MM (*n*=7) or MGUS patients with long-term stable disease (no progression for 5 years or more; *n*=13). Data from the MM cohort (*n*=9) presented in Figure 1 is replicated here for comparison purposes. Box and whisker plots depict median and interquartile ranges. *p*-values are shown for Kruskal-Wallis test with Dunn's post-test.

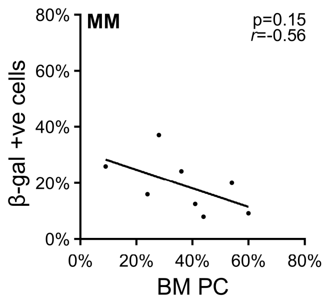

Supplementary Figure 3 MSC senescence in MM patients is not associated with tumour burden. Scatter dot plot showing correlation between % β-gal positivity in MSCs and bone marrow PC burden in MM patients. *p*-value is shown for Pearson's correlation.

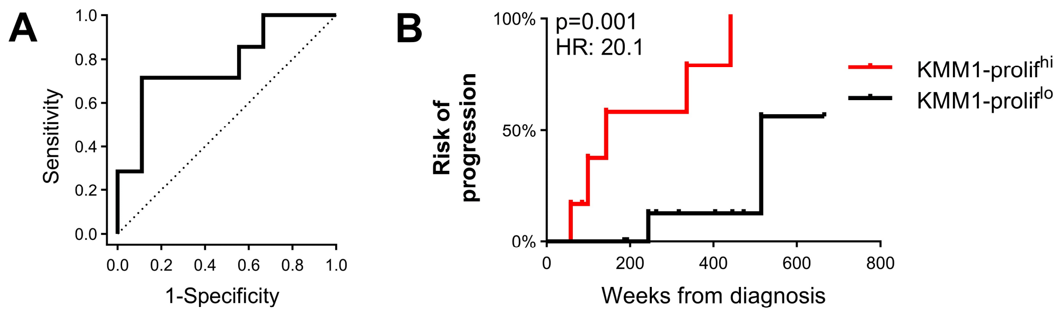

Supplementary Figure 4 MSC ability to support KMM1 growth *in vitro* is associated with increased risk of MGUS to MM progression. A. ROC analysis of the sensitivity and specificity of MSC effects on KMM1 cell proliferation in vitro on MGUS to MM progression. **B.** MGUS patients stratified on MSCs having a greater suppressive effect on KMM1 cell proliferation in vitro (KMM1-prolif^{lo}; <69% of proliferation level observed in KMM1 monoculture, n=17) and those that had less capacity to suppress KMM1 proliferation (KMM1-prolif^{hi}; ≥69% of proliferation observed in KMM1 monoculture, n=9) were assessed for rate of MGUS to MM progression using Kaplan-Meier analysis. Kaplan-Meier curves were compared with the log-rank test.

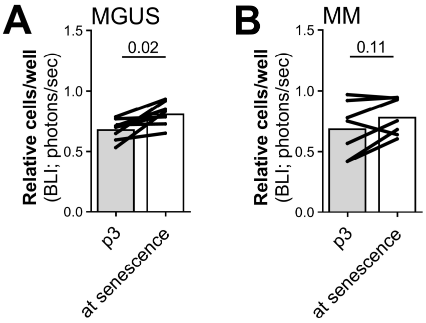

Supplementary Figure 5 Replication-induced MSC senescence alleviates MSC-mediated suppression of MM cell proliferation in vitro in MSCs isolated from MGUS and MM patients. Human MSCs cultures, isolated from MGUS patients (**A**) or MM patients (**B**) were passaged twice weekly until they reached replicative senescence (MGUS: passage 4-13; MM: passage 5-18). Passage 3 and replicatively senescent MSCs were seeded, allowed to adhere overnight and coculture was initiated with luciferase-expressing KMM1 cells. After 3 days, the relative number of KMM1 cells was enumerated using BLI. Graphs show paired values for 6 independent donors/group, bar represents mean. p-values are shown for paired *t*-test.

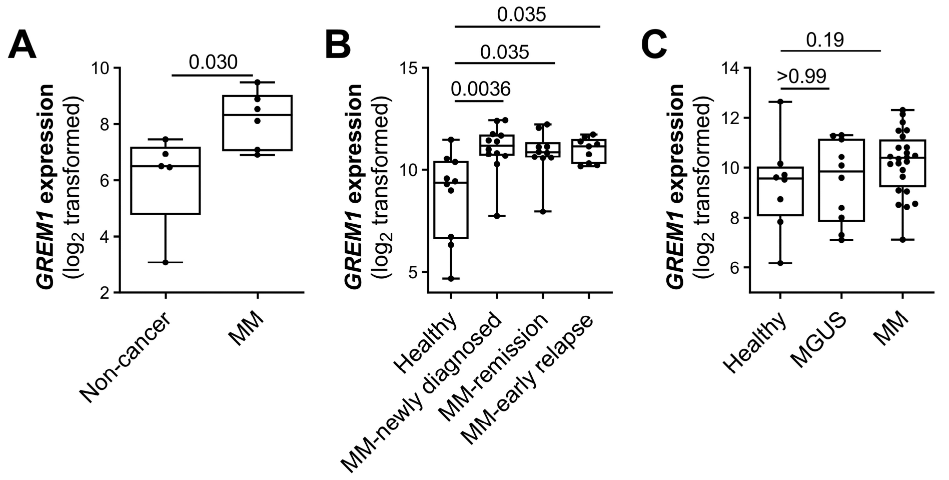

Supplementary Figure 6 Gremlin1 expression is consistently upregulated in MM MSC when compared to those of non-myeloma or healthy controls. A. *GREM1* expression, as assessed by RNA sequencing, in BM MSCs from MM patients (*n*=6), and non-myeloma controls (*n*=5) in GEO dataset GSE196297. **B.** *GREM1* expression, assessed by Affymetrix Human Genome U133 Plus 2.0 Array, in BM MSCs from newly diagnosed MM patients (*n*=10), newly diagnosed MM patients in complete remission (*n*=10), MM patients in early relapse (*n*=9) and healthy controls (*n*=10) in dataset GSE146649. **C**. *GREM1* expression, assessed by Affymetrix Human Gene 1.0 ST Array, in BM MSCs from MM patients (*n*=24), MGUS patients (*n*=24) and healthy controls (*n*=8) in dataset GSE137369. All raw data were processed and $\log_2$ transformed using GEO2R. Box and whisker plots depict median and interquartile ranges. *p*-values are shown for Mann-Whitney test (**A**) or Kruskal-Wallis test with Dunn's post-test (**B**-**C**).
